# Screening for colorectal cancer: possible improvements by risk assessment evaluation?

**DOI:** 10.3109/00365521.2011.610002

**Published:** 2011-08-19

**Authors:** Hans J Nielsen, Karen V Jakobsen, IB J Christensen, Nils Brünner

**Affiliations:** 1 Department of Surgical Gastroenterology, Hvidovre Hospital, Hvidovre, Denmark; 2 Faculty of Medicine and Surgery, University of Copenhagen, Hvidovre, Denmark; 3 The Finsen Laboratory, Copenhagen Biocenter, Copenhagen, Denmark; 4 Department of Veterinary Disease Biology, Faculty of Life Sciences, University of Copenhagen, Frederiksberg, Denmark; 5 Appendix

**Keywords:** biomarkers, colorectal cancer, oncology-clinical, risk evaluation, screening

## Abstract

Emerging results indicate that screening improves survival of patients with colorectal cancer. Therefore, screening programs are already implemented or are being considered for implementation in Asia, Europe and North America. At present, a great variety of screening methods are available including colono- and sigmoidoscopy, CT- and MR-colonography, capsule endoscopy, DNA and occult blood in feces, and so on. The pros and cons of the various tests, including economic issues, are debated. Although a plethora of evaluated and validated tests even with high specificities and reasonable sensitivities are available, an international consensus on screening procedures is still not established. The rather limited compliance in present screening procedures is a significant drawback. Furthermore, some of the procedures are costly and, therefore, selection methods for these procedures are needed. Current research into improvements of screening for colorectal cancer includes blood-based biological markers, such as proteins, DNA and RNA in combination with various demographically and clinically parameters into a “risk assessment evaluation” (RAE) test. It is assumed that such a test may lead to higher acceptance among the screening populations, and thereby improve the compliances. Furthermore, the involvement of the media, including social media, may add even more individuals to the screening programs. Implementation of validated RAE and progressively improved screening methods may reform the cost/benefit of screening procedures for colorectal cancer. Therefore, results of present research, validating RAE tests, are awaited with interest.

Colorectal cancer (CRC) represents a major public health problem accounting for more than one million new cases and approximately half a million deaths worldwide every year [1]. At primary diagnosis 80% of the patients will undergo intended curative resection, but 40-45% of these patients will develop recurrent disease within the next 5 years [2,3], most often leading to fatal outcome. Presently, at the time of diagnosis half of the patients have stage I or II disease, and the other half have disseminated disease, stage III or IV. It is assumed that overall survival would be substantially improved if more patients were detected and diagnosed at an early stage [4,5]. Such a hypothesis is supported by the fact that population screening for CRC using fecal occult blood test (FOBT) results in more individuals being diagnosed with an early-stage disease [6,7], which translates into improved survival compared to CRC cases not detected by screening [8-10]. Recent reports suggest that screening procedures may even reduce the incidence of CRC due to detection and removal of precancerous lesions [11,12]. Therefore, general screening procedures for CRC are shown to reduce incidence and improve survival [13,14].

## Justifying screening for CRC

The following factors justify screening for CRC:

- The disease causes significant morbidity and mortality
- Treatment of the disease is a financial burden for the society
- The disease is detectable in an asymptomatic stage
- The disease can be prevented with intervention at the premalignant stage
- Effective treatment modalities are present for early stages
- Higher survival rates among patients detected by screening
- Early detection carries benefits for the patient
- Early detection carries benefits for the society

## Who should be offered screening?

The incidence of CRC is age-related with a median age of 70 years [15] and the lower quartile at the age of 58. Current studies of screening and implemented screening programs for the average risk population include, with few exceptions [16], individuals in the age range of 50-75 years [17]. It is currently suggested that screening offers must be extended to individuals between 40 and 85 years of age [18], due to findings that significant proportions of individuals between 40 and 50 years have large bowel neoplastic lesions, combined with the fact that overall lifetime expectancy is increasing. Feasibility and subsequent success in screening programs may lead to debate on the suggested extension of the hitherto agreed age intervals.

## Present methods for CRC screening

Colonoscopy is still considered the gold standard for detection of neoplastic large bowel lesions. The procedure allows examination of the entire large bowel with ability to biopsy lesions and even to remove most adenomas within the same sequence. The procedure is used either as an examination without any pretest, or in combination with FOBT and/or flexible sigmoidoscopy. Colonoscopy is also used subsequent to capsule-, CT- or MR-colonography, where detected lesions can be excised colonoscopically.

### Direct colonoscopy

The procedure is widely used for screening particularly in the USA. Due to improvements in the US Medicare system the number of individuals screened by colonoscopy without any pretest has increased since 2008 [19]. Improved referral programs among primary care practitioners in the USA have led to procedures where low-risk individuals might undergo direct colonoscopy without the need for a preconsultation visit at the endoscopists' clinic [19]. However, direct colonoscopy is still an expensive screening procedure: First, colonoscopy requires complete bowel preparation, which must be initiated 2-3 days before the procedure. Second, current medications must be taken into account among some individuals whereas patients with diabetes need specific attention and preparation; some may even be hospitalized. Third, at least one day off work — the day before a colonoscopy procedure - may be expected due to bowel evacuation. Furthermore, most individuals need sedation during colonoscopy, which leads to an additional half or whole day off work, and often a third person has to take care of the transport back from the clinic. In total, an individual who undergoes colonoscopy is completely out of work or daily routines for 1.5-2 days. The sum of all these costs added to the costs of the colonoscopy procedure causes major expenses [20]. Finally, colonoscopy may lead to adverse events such as cardiopulmonary incidents (1.1%), bleeding (0.16%), perforation (0.09%) and death (0.007%) [21].

Although screening by colonoscopy may secure detection of the majority of all bowel lesions, the procedure is still not an option for general population screening because of the fact that no society has either the capacity or the financial resources to offer the procedure to all its citizens. The costs of the procedure are approximately 1000-1200 USD per individual, even without inclusion of the costs of recovery and 1.5-2 days off work or daily routines. Adverse events add even more costs to the procedure. Offering colonoscopy to individuals between 50 and 75 years of age, for instance, every 10th year would require an examination capacity for about 250 million, individuals in Europe and North America alone. Thus, the cost-benefit of a model primarily based on colonoscopy has to get significantly improved.

### Colonography and capsule endoscopy

CT-colonography (CTC) could be an attractive, non-invasive tool to visualize the entire bowel and thereby identify lesions in the bowel wall. Obviously, this approach may be of advantage among persons where colonoscopy is not feasible either due to personal resistance or due to known diverticulosis, where passage even with a thin colonoscope is cumbersome. An additional advantage of CTC is detection of extracolonic lesions [22,23]. However, CTC also has its disadvantages; the procedure still requires meticulous bowel evacuation and air insufflation of the bowel. In addition, lesions <6 mm, flat adenomas or adenocarcinomas appear to be missed [24]. Finally detected lesions require subsequent colonoscopy or sigmoidoscopy for excision purposes. Moreover, the CTC-associated radiation burden is high and should be considered among the disadvantages of the procedure, particularly among persons undergoing repeated screening procedures. Even detection of extracolonic lesions may be of potential disadvantages, because such lesions may add further examinations to the ongoing procedures. Most of such lesions turn out to be benign and do not carry any significant risks [22].

Whether CTC would increase the compliance rates in screening programs compared with colonoscopy is presently unknown; also, the cost-effectiveness of CTC is yet to be established. The aims in a current Dutch study on 7,500 persons in the age range of 50-75 years include compliance, yield and costs. The persons are randomized to either CTC (one-third) or colonoscopy (two-thirds), and, in addition, the need for pre-endoscopy consultations is also studied [25]. A recent report, though, based on models using four different technologies, namely, FOBT, flexible sigmoidoscopy, colonoscopy and CTC, have estimated lifetime costs and outcomes of a cohort of persons screened at 60-69 years of age [26]. The report concluded that the use of CTC every 10th year has a potential to be as cost-effective as biennial FOBT screening [26]. However, these results need to be verified in sufficiently powered clinical trials before CTC is considered a preferable procedure in screening for CRC.

Compared to CTC, MR-colonography (MRC), which is also a non-invasive procedure, may have the advantage of being free from the risk of radiation damages. Furthermore, new techniques with fecal tagging have significantly reduced the need for bowel evacuation [27,28], and also the acceptability seems to be an advantage of MRC compared to colonoscopy [28]. It appears, though, that MRC technology may not detect lesions <6-10 mm [29], and a colonoscopy will still be required in most cases of detected lesions. Additional research is urgently needed to evaluate the cost-effectiveness of the MRC procedure as general population screening. Presently, it is not likely that MRC will be recommended for screening.

Endoscopy using bowel cameras in large capsules might be a future option, and the yield may be comparable with CTC and MRC. However, meticulous bowel evacuation and subsequent colonoscopy or flexible sigmoidoscopy will also be needed in those persons where lesions are detected. Still sufficiently powered studies are needed to evaluate this approach in comparison with other presently available and accepted procedures.

### Flexible sigmoidoscopy

Approximately 70-75% of the neoplastic large bowel lesions are located in the rectum and left colon. The bowel preparation for sigmoidoscopy is significantly less compared with the preparation for colonoscopy: Rigid fast of solid foods from midnight the day before sigmoidoscopy and a small cleansing enema taken 1-2 h before sigmoidoscopy often sufficiently leads to evacuation of the left-sided bowel. These facts justify approaches for screening procedures using flexible sigmoidoscopy. The fact that this procedure does not require any pre-test may lead to a high patient acceptance and thereby an acceptable compliance [30,31]. The approach mediates reduction of the incidence and mortality of CRC [30,31], particularly of the left-sided tumors. Although a UK-based randomized study [31] showed reduction of mortality due to tumors anywhere in the colonic bowel, most studies have shown that transverse and right-sided lesions are missed [32-34] even when combined with a subsequent FOBT [32]. These results plus those from a Norwegian study [30] raise the question of whether sigmoidoscopy should be recommended in place of colonoscopy for future screening procedures, or at least considered an option comparable to colonoscopy for individuals offered screening for CRC [35-37]. A recent Canadian case-control study showed that colonoscopy led to reduction in mortality of left-sided lesions, but similar reduction could not be shown for right-sided lesions [38]. Such results may add power to sigmoidoscopy, but subsequent discussion awaits results from current randomized studies, including those on colonoscopy, showing whether the supposed benefits of the sigmoidoscopy procedure can be confirmed [37].

### Fecal occult blood tests

FOBT screening has shown its ability to reduce mortality resulting from CRC [6-11] and several countries have implemented or are considering implementing the procedure for general population screenings. The specificity of the various tests is around 85-90%, while the sensitivity of the tests has a wide range of 40-90%. Results from a variety of studies have shown, however, that compliance varies between 30% and 85%. These figures are notable, because lack of compliance leads to significantly limited clinical sensitivity (test sensitivity X compliance), that is, between 12% and 76%. Consequently, a significant number of bowel lesions will not be detected. Therefore, exploration of the mechanisms leading to rejection of the offer of FOBT screening is needed in order to improve the procedure. Otherwise investments in such screening programs may be unsuccessful and turn out to have limited value.

In general there are two different FOBT tests: the guaiac (G-FOBT) and the immunologic (FIT). Patient preparation for the G-FOBT is extensive and includes a variety of drugs and diets that must be omitted. Thus, for a period of 7 days before the stool-sampling period, patients must not use NSAIDs and many other similar drugs, because these may lead to false positive results of the G-FOBT. Also, 3 days before stool-sampling, patients should avoid red meat (lamb, beef and liver), and 24 h before the test sampling, they must refrain from alcohol, aspirin and vitamin C [11]. Next step -stool-sampling - may be a complicated maneuver for many persons; the procedure requires the individual to deal with his or her feces in three consecutive collections. The samples must be transferred to the provided cards, dried and mailed. If the test results are positive, the patient is assumed to be at risk of having a neoplastic lesion in the large bowel and is, therefore, offered subsequent colonoscopy and/or other complete diagnostic evaluation procedures. Surprisingly, many such at-risk persons are not willing to undergo diagnostic evaluation, including colonoscopy (in some studies 40-65%). Mere patient refusal, high age, young age or persons without insurances often explain the refusals; lack of insurance is a particular limiting phenomenon in the US [39-41].

Nevertheless, emerging results have shown that the number of patients detected with stage I or II diseases by FOBT is significantly increased, while the number with stage IV diseases is decreased [11]. Thus in a recent Danish feasibility study [42] with 177,148 persons between 50 and 74 years of age, 85,374 persons returned their test card to the study centers (compliance 48.2%). Positive test results were shown in 2,085 persons, who were offered subsequent colonoscopy. Among these, polyps were detected in 841 and CRC in 174 persons (ppv for CRC = 8.3%). The CRC diagnoses among the screened persons were stage I: 36.8% [Danish Colorectal Cancer Group (DCCG) data on all Danish CRC cases [43] was 12.5%]; stage II: 27.6% (DCCG data, 33.2%); stage III: 27.0% (DCCG data, 28.6%); and stage IV: 8.0% (DCCG data, 21.2%). These data from the Danish feasibility study [42] are similar to data from other international screening protocols and indicate that screening substantially identifies cancer patients at an early stage, and through this improves the chances for complete resection of more patients than in non-screening populations [11,44-45].

Introduction of the FIT test has led to an improved pre-sampling preparation procedure. Using enzymatic reactions [46] the test was developed to detect human hemoglobin, and thereby the need for dietary restrictions (i.e., red meat and various drugs) before stool-sampling was performed [47]. Therefore, as expected [11], both compliance and ppv and npv rates were substantially improved [48], particularly for CRC [46] but also for adenomas [49,50]. Results of FIT screening procedures and subsequent colonoscopy on mortality have yet not been reported, but are awaited with interest. Comparisons of performances of G-FOBT and FIT have demonstrated an increased sensitivity of FIT both for CRC and adenoma detection [48,49,51-52]. If FIT screening procedures improve the mortality of CRC, these particular tests should be recommended for future screenings for adenoma and CRC instead of G-FOBT screening.

### Fecal DNA tests

Development of neoplastic lesions in the large bowel appears to be based on hereditary genetic syndromes in 5-10% of the cases, while sporadic neoplastic lesions based on gene mutations appear in 90-95% of the cases. The progression from precursor lesions over benign to malignant lesions seems to be due to sequential genetic changes [53]. Because of exfoliation, cellular elements containing genetic information on these genetic changes are mixed and shed with the stools. This observation led to development of various assays for DNA identification procedures in stools, and the procedure appears to be a feasible option for detection of neoplastic lesions in screening for CRC [54,55]. However, the accuracy of the test does seem comparable to the G-FOBT tests. The costs, though, are 30-fold higher than the costs for G-FOBT and 10-fold higher than those for FIT [11]. In addition to the costs, the stool-sampling procedures are needed to be focused upon as well. Stool-sampling for the DNA tests includes a plastic bucket that is mounted onto the toilet. The whole bowel excretion must be collected without contamination by urine or toilet paper. The bucket is subsequently transferred to a cooling device and shipped by mail to a given reference laboratory. Once in the laboratory stools are homogenized and treated with RNase, followed by purification and real-time polymerase chain reaction using target specific primers, all of which is a rather complicated procedure. Therefore, DNA stool testing is expected to have only limited impact on overall future screening procedures.

## Rationale for new test methods

At present the variety of tests and procedures for screening for CRC appears challenging for the health authorities and in particular for the health budgets. General implementation of the gold standard - colonoscopy - for all persons between 50 and 74 years of age would require a capacity to screen 250 million, individuals over a 10-year period in Europe and North America. The frequency would certainly be increased in those persons with precancerous lesions, and thereby more individuals must undergo colonoscopy per year. As no nation would have such resources and capacity available, we need to focus on selection procedures (prescreening) for colonoscopy, which could, for instance, be tests for occult blood in feces. Among these tests, the results by FIT screening are compelling. Using FIT screenings may be the definitive choice, if emerging results of using this model show reduced mortality by CRC. In spite of high specificities and fair sensitivities in all available tests for occult blood and DNA in feces [11], the limited compliance rates may be the major hindrance in using such tests for present and future general screening procedures. Often compliance rates are as low as between 40% and 50%. Thereby the clinical sensitivity is substantially reduced leaving many persons with unknown neoplastic lesions.

Refusal of participation in prescreening procedures and subsequently in colonoscopy among a substantial number of persons with positive FOBT or FIT tests may lead to considerations of at least three alternatives: (1) offering other diagnostic tests such as, for instance, MR- or CT-colography; (2) development of a prescreening tests with improved positive predictive value (ppv) and negative (n)pv and/or; (3) major media campaigns focusing on the subject. The American Society of Colon and Rectal Surgeons have recently recognized the significance of proper media coverage on CRC screening by giving awards to three reporters [56] who made a substantial effort in covering the subject.

## Future prescreening test options

Among future options for screening procedures are implementation of blood tests for proteins and genes related to CRC and possibly to adenomas. Carcinoembryonic antigen (CEA) in serum was the first soluble biomarker accepted for use in CRC and is still the only recommended soluble biomarker being primarily used for monitoring purposes. However, the level of CEA is strongly dependent of the stage of disease with a low positive rate in early-stage disease and a high positive rate in late-stage disease.

Therefore, the sensitivity of CEA in screening varies between 8% and 89% at specificities of 70-95% [57]. The latest American Society for Clinical Oncology (ASCO) guidelines accordingly recommend that CEA is not used as a single screening test for CRC [58].

Plasma tissue inhibitor of metalloproteinases-1 (TIMP-1) has been suggested for early detection of CRC [59-61], as high plasma TIMP-1 levels were shown to identify colon cancer (CC) patients with a sensitivity of 63% at 98% specificity, patients with early CC (stage I and II) with a sensitivity of 56% at 98% specificity and patients with right-sided CC with a sensitivity of 72% at 98% specificity [61]. The rates for detection of RC are not as prominent. A subsequent study by independent investigators supported that the plasma TIMP-1 protein level may be an important marker in early detection of CRC showing 42% sensitivity at 95% specificity [62]. Of specific interest was that the discrimination was significantly improved by combining TIMP-1 with CEA measurements [61]. The previous studies used retrospectively collected plasma samples from patients with known CRC and healthy blood donors as control individuals. Such approaches may introduce bias since blood donors are not representative of a CRC-related background population. Another potential confounder in such studies is that samples from patient cohorts and healthy volunteers may not be collected simultaneously and, therefore, often not be according to similar standard operating procedures (SOPs).

The tumor marker utility grading system (TMUGS) guidelines [63] suggest that retrospectively obtained results must be prospectively validated in order for a biomarker to reach clinical acceptance and subsequent implementation. Such prospective studies should take all possible pre-, intra- and post-analytical aspects into consideration, including the use of strict and identical sampling, handling and storage procedures for specimens from all recruited individuals [64].

Thus a prospective, population-based validation study including individuals scheduled for large bowel endoscopy due to symptoms of CRC were initiated [65]. The primary aim of the study, which included 4,509 individuals, was to validate the combination of plasma TIMP-1 and CEA as biomarkers in early detection of CRC. Overall the results supported the fact that the combination of plasma TIMP-1 and CEA was a valuable biomarker in early detection of the disease, specifically of CC [66]. It was also demonstrated that both plasma TIMP-1 and CEA levels were significantly increased in individuals without CRC, but diagnosed with a variety of non-malignant diseases including diabetes I or II, bronchitis, asthma, chronic obstructive lung diseases, and various cardiovascular and liver diseases [66]. The compliance of the study was 96.8%; it should be kept in mind however, that the individuals had symptoms of CRC and as such were at-risk individuals, who were admitted for examination of the large bowel.

Subgroups of samples were used to identify possible new biomarkers that might be used for early detection of CRC either as single markers or in various combinations of markers. One group included 77 samples collected from individuals who turned out to have CRC, and a second group consisted of 77 samples from age- and gender-matched individuals with adenoma at the same location as the CRC lesions. A third group were 77 samples collected from individuals with other non-malignant GI findings (diverticulosis) and the fourth group included samples from 77 individuals with no findings and no concurrent disease (*n* = 308 in total). At present, the liberated domain I of the urokinase receptor suPAR(I) in plasma appears to have the potential as a biomarker in CRC [67-69] and might be useful in early detection [67]. Results from a variety of other potential biomarkers, including proteins, miRNAs, and SNPs, are awaited.

All achieved results from studies of plasma TIMP-1 and CEA [61,62,66] are comparable with the emerging results of *Septin 9* (SEPT9) regarding sensitivity and specificity for CRC detection. SEPT9 determinations represent a marker of hypermethylation in DNA extracted from plasma collected from patients with known CRC and plasma from a variety of healthy and non-cancerous control individuals [70,71]. Results with SEPT9 have very recently been confirmed in a population-based, prospective study on 7,940 individuals admitted to screening for CRC [72]. In this particular study all individuals had plasma collected before colonoscopy, which identified 53 individuals with CRC. The sensitivity of the SEPT9 test was 66.7% at 88.4% specificity. The company behind the test suggests that the SEPT9 test is the future prescreening test, and that screened persons may be very confident with a negative result of the test due to its npv of 99.7% [72]. As with plasma TIMP-1 and CEA, SEPT9 also identifies individuals with a variety of other diseases than CRC, a fact that must be considered also among CRC patients, who may have one or more of these diseases in addition to CRC. A future option might be to evaluate the combination of plasma SEPT9 with plasma TIMP-1 and CEA. Such combined results may lead to a higher performance in detection of CRC and possibly also precancerous lesions among individuals without symptoms [66,73].

Summarizing all the facts presented here leads to the conclusion that no single test appears to have an acceptable ppv, and the various tests identify individuals with diseases other than CRC or adenoma. Therefore, a usable option could be to establish a risk assessment evaluation (RAE) test. Such a test is to be based on demographic and clinical parameters [74] in combination with various blood tests of proteins, gene polymorphisms, methylated genes, microRNAs and so on. It is well known that age, gender, race and BMI, plus a variety of diseases, carry a higher risk of developing CRC ([75-98], Table I). Alcohol consumption has been suggested as an additional risk factor [95], but recent results could not support this suggestion [99-101]. Therefore, alcohol consumption should presently not be included in a RAE test.

**Table I:** Increased risk of developing colorectal neoplasia is associated with the following factors

Age	75,76
Gender	77,78
Race	79,80
BMI	81,82
Smoking habits	78,83,84,85
Inflammatory bowel disease	86,87,88
Diabetes type II	89,90,91,92,93
Familial disposition	94,95
Hereditary disposition	96,97,98

## Possible RAE model

**Figure 1:**
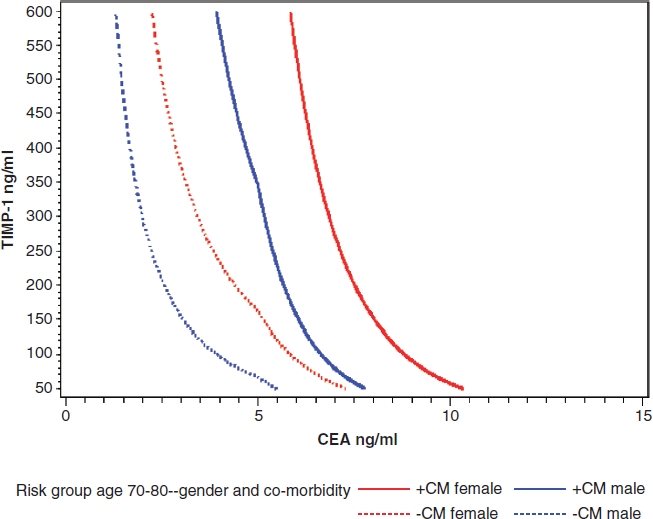
A male without co-morbidity with plasma TIMP-1 and CEA co-ordinates to the right of the dotted blue line would be considered positive for this test. Similarly, a male with co-morbidity with plasma TIMP-1 and CEA co-ordinates to the right of the solid blue line would be positive for the test. A female with or without co-morbidity and with plasma TIMP-1 and CEA co-ordinates to the right of the solid red or the dotted red line, respectively, would be considered positive for the test. CEA: Carcinoembryonic antigen; TIMP-1: Tissue inhibitor of metalloproteinases-1.

Figure 1 represents a RAE based on the results from the recent, aforementioned study on early detection of CRC in a high-risk population [66]. The study showed that age and gender were independent predictors of CRC, and that comorbidities influenced the significance of both plasma TIMP-1 and CEA levels. For example, plasma TIMP-1 levels as well as plasma CEA levels in an individual with co-morbidity will often be higher than in an individual without co-morbidity. The exampled normogram based on such tests for individuals aged 70-80 years adjusted for gender and co-morbidity is shown in Figure 1. Choosing a probability of 15% for CRC as a cut-point in this high-risk population, a test based on these covariates had a specificity of 94% with a sensitivity of 43%. A male without co-morbidity with plasma TIMP-1 and CEA coordinates to the right of the dotted blue line would be considered positive for this RAE test. Similarly, a male with co-morbidity would only be considered positive for this RAE test if the co-ordinates of his plasma TIMP-1 and CEA levels fall to the right of the solid blue line. A female with or without co-morbidity and with plasma TIMP-1 and CEA coordinates to the right of the solid red or the dotted red line, respectively, would be considered positive for the test. Conclusively, individuals with positive test results must be offered colonoscopy for further diagnostic purposes. Thus using this RAE test can be beneficial as a prescreening and selection test.

It is urgent to identify valid biological markers with high ppvs and npvs to be included into a RAE test. At present, a major (>5,000 individuals) study on validating the suggested RAE combined with demographic, clinical and biomarker parameters is being performed across Denmark. Simultaneously, various biomarkers are identified [73,102-110] and must be evaluated and validated to be included into the RAE. In the event that the RAE passes this validation, new biomarkers can be included, whenever they pass the TMUGS guidelines [63]. Thereby the RAE test can be progressively improved [111].

There are several benefits of instituting RAE tests for prescreening of CRC. First of all this test can be completed whenever needed. When a single individual in conjunction with his or her practitioner has performed the basic RAE test once, the subsequent calculation could be performed online. Next time the individual only has to leave a blood sample for testing, and the subsequent results can be added into the online calculation sheet. Changes in scores will then work as a guide for further examinations including colonoscopy. Second, by using an easily available prescreening test as the RAE test, compliance can be improved substantially through supportive media campaigns, even using electronically available technology such as social medias, automated telephone calls and text messages [112,113]. Moreover, the fact that all individuals follow their risk scores on their own might reduce the refrain from undergoing subsequent colonoscopy [39-41]. In cases where individuals still refuse to undergo colonoscopy, they might be offered either a subsequent FIT test to further encourage colonoscopy or direct CT- or MR-colonography with subsequent colonoscopy/resection, if lesions are detected.

It has been argued, however, that screening procedures may lead to anxiety among some individuals, particularly when results are awaited over days. But results from a recent population-based study on psychological distress following FOBT screening could not demonstrate any adverse effect on psychological well-being [114].

In summary, we are confident that CRC screening can be appreciably improved. Particularly, an implementation of validated RAEs might improve compliance and thereby lead more individuals to a subsequent large bowel examination. Therefore, the results of ongoing studies in Europe and USA are awaited with interest.
